# Microbiological study of ventilator-associated pneumonia (VAP) after implantation of pneumonia program zero (NZ)

**DOI:** 10.1186/2197-425X-3-S1-A1021

**Published:** 2015-10-01

**Authors:** R Fernández Fernández, MR Ramírez Puerta, ME Yuste Ossorio, O Moreno Romero, M Muñoz Garach

**Affiliations:** Hospital Universitario San Cecilio, Unidad Cuidados Intensivos, Granada, Spain

## Introduction

The National Survey of Nosocomial Infection Surveillance (ENVIN) is a computerized record of the incidence of nosocomial infection in ICU in Spain. In our hospital, we have drafted rules for the maintenance of the airway in mechanically ventilated patients following general recommendations.

## Objectives

Describe the clinical diagnosis and microbiology of VAP after the implementation of the NZ program.

## Methods

18 ICU beds. Data from ENVIN 01.04.11 / 01.04.15 (clinical diagnosis, microorganisms isolated and antibiotic resistance, inflammatory response ).

NZ: Educational campaign (presencial and by means of internet) to professionals of our unit and anesthesia-resuscitation unit (112 professionals). Were instituted mandatory measures for the prevention of VAP.

## Results

**01.04.11/01.04.12**- 15 VAP.

- Clinical diagnosis:

support clinical radiological+new infiltrate 80%.

Extension of previous infiltrate + clinical worsening (2nd pneumonia)20%

- Germ:

A. baumanii 44.44%: resistance: amikacin, ampicilin, imipenem, tobramicin

P. aeuroginosa 22.22%: resistance: amikacin, ceftacidim, ciprofloxacin,

colistin, imipenem, levofloxacin, meropenem, piperacilina-tazobactam (P-T)

MRSA 16.67%

Polymicrobial 26.67%.

- Inflammatory response: sepsis 33.33%, severe sepsis 33.33%.

**01.04.12/01.04.13-**6 VAP

- Diagn:

support clinical radiological+new infiltrate 66.67%

extension of previous infiltrate + clinical worsening (2nd pneumonia) 16.67%,

CT 16.67%.

- Germ:

S. Aureus 22 .22%: no resistance

S. Maltophilla 22.22% no resistance

Polymicrobial 50%.

Without diagnostic 16.67%

- Inflammatory response: sepsis 50%, severe sepsis 33.33%, shock 16.67%

**01.04.13/01.04.14**- 10 VAP

- Diagn:

support clinical radiological+new infiltrate 60%

extension of previous infiltrate + clinical worsening (2nd pneumonia) 30%,

CT 10%.

- Germ:

S. Aureus 25%

E. Coli 16.67%: resistance: ciprofloxacin, amox-clav.

H. Influnzae 8.33%

P. aeuroginosa 8.33%: resistance :cefepime, ceftacidime, imipenem,

meropenem, P-T

polimicrobial 20%.

- Inflammatory response: sepsis 50%, severe sepsis 30%, shock 20%.

**01.04.14/01.04.15**- 3VAP

- Diagn:

support clinical radiological+new infiltrate 68.57%

extension of previous infiltrate + clinical worsening (2nd pneumonia) 22.86%

CT 8.57%.

- Germ:

A. baumanii 18.6% . Resistance: amikacin

P. aeuroginosa 16.28% . Resistance: amikacin, cefepime

S. aureus 13.95%: no resistance

Polimicrobial 28.57%

- Inflammatory response: sepsis 33.33%, severe sepsis 66.67%

## Conclusions

Most of the diagnoses are made by the patient's clinical and x-ray. Each year vary germs being the most frequent S. aureus and P. aeruginosa. Usually, the most common inflammatory response is sepsis. The P. aeruginosa is the germ which has more resistance.

## Grant Acknowledgment

UCI S. Cecilio
